# Prior thermal acclimation gives White Sturgeon a fin up dealing with low oxygen

**DOI:** 10.1093/conphys/coae089

**Published:** 2025-01-06

**Authors:** Angelina M Dichiera, Kelly D Hannan, Garfield T Kwan, Nann A Fangue, Patricia M Schulte, Colin J Brauner

**Affiliations:** Department of Zoology, The University of British Columbia, Vancouver, British Columbia, Canada; Department of Wildlife, Fish and Conservation Biology, University of California Davis, Davis, California, USA; Department of Wildlife, Fish and Conservation Biology, University of California Davis, Davis, California, USA; Department of Wildlife, Fish and Conservation Biology, University of California Davis, Davis, California, USA; Department of Zoology, The University of British Columbia, Vancouver, British Columbia, Canada; Department of Zoology, The University of British Columbia, Vancouver, British Columbia, Canada

**Keywords:** multiple stressors, global climate change, fish, tolerance, temperature, hypoxia

## Abstract

Assessing how at-risk species respond to co-occurring stressors is critical for predicting climate change vulnerability. In this study, we characterized how young-of-the-year White Sturgeon (*Acipenser transmontanus*) cope with warming and low oxygen (hypoxia) and investigated whether prior exposure to one stressor may improve the tolerance to a subsequent stressor through “cross-tolerance”. Fish were acclimated to five temperatures within their natural range (14-22°C) for one month prior to assessment of thermal tolerance (critical thermal maxima, CTmax) and hypoxia tolerance (incipient lethal oxygen saturation, ILOS; tested at 20°C). White Sturgeon showed a high capacity for thermal acclimation, linearly increasing thermal tolerance with increasing acclimation temperature (slope = 0.55, adjusted R^2^ = 0.79), and an overall acclimation response ratio (ARR) of 0.58, from 14°C (CTmax = 29.4 ± 0.2°C, mean ± S.E.M.) to 22°C (CTmax = 34.1 ± 0.2°C). Acute warming most negatively impacted hypoxia tolerance in 14°C-acclimated fish (ILOS = 15.79 ± 0.74% air saturation), but prior acclimation to 20°C conferred the greatest hypoxia tolerance at this temperature (ILOS = 2.60 ± 1.74% air saturation). Interestingly, individuals that had been previously tested for thermal tolerance had lower hypoxia tolerance than naïve fish that had no prior testing. This was particularly apparent for hypoxia-tolerant 20°C-acclimated fish, whereas naïve fish persisted the entire 15-h duration of the hypoxia trial and did not lose equilibrium at air saturation levels below 20%. Warm-acclimated fish demonstrated significantly smaller relative ventricular mass, indicating potential changes to tissue oxygen delivery, but no other changes to red blood cell characteristics and somatic indices. These data suggest young-of-the-year White Sturgeon are resilient to warming and hypoxia, but the order in which these stressors are experienced and whether exposures are acute or chronic may have important effects on phenotype.

## Introduction

Globally, human impacts are changing the environment and exposing organisms to a range of stressors that vary in magnitude and across timescales. Not only are global temperatures rising (IPCC, 2023), but extreme heat events are also increasing in frequency and duration (Meehl and Tebaldi, 2004; Oliver *et al.,* 2018). Both rapid and prolonged changes to thermal regimes threaten ectothermic animals, impacting fitness and distribution (Fry, 1967; Deutsch *et al.,* 2008; Sunday *et al.,* 2011; Sunday *et al.,* 2012; Sunday *et al.,* 2019; Dahms and Killen, 2023). In addition, these thermal challenges often co-occur with other environmental stressors including decreased oxygen availability (hypoxia; Breitburg *et al.,* 2018; Rabalais *et al.,* 2010), which is thought to drive global distributions of aerobic organisms (Deutsch *et al.,* 2015; Penn *et al.,* 2018; Deutsch *et al.,* 2020; Gallo *et al.,* 2020). Thus, for ectothermic animals, the combined stressors of warming and hypoxia are intricately linked and potentially physiologically costly (Earhart *et al.,* 2022; McBryan *et al.,* 2013; Del Rio *et al*., 2021).

In fishes, vulnerability to low oxygen availability tends to increase when combined with warming (Vaquer-Sunyer and Duarte, 2011; Rogers *et al.,* 2016; Earhart *et al.,* 2022). This may be driven at least in part by higher metabolic cost and resulting increased oxygen consumption at warmer temperatures. For some species, prior exposure to one stressor may improve the tolerance to a subsequent stressor through “cross-tolerance”. Acclimation to warm temperatures has been shown to improve hypoxia tolerance in salmonids (Anttila *et al.,* 2015), and species such as red drum (*Sciaenops ocellatus*; Zambie *et al.,* 2024), common carp (*Cyprinus carpio*; Opinion *et al.,* 2021), and killifish (*Fundulus heteroclitus*; McBryan *et al.,* 2016) which are known for their impressive phenotypic plasticity. However, in general, fishes only partially compensate for the negative effects of acute warming and hypoxia (reviewed by Earhart *et al.,* 2022), and in some cases, the acclimation response is insufficient to combat the negative effects of the combined stressors. For example, Amazonian fishes demonstrated reduced hypoxia tolerance with both acute and long-term exposures to warmer temperature (Jung *et al.,* 2020). Similarly, acute high temperature exposure reduces hypoxia tolerance in coral reef fishes and these effects are not mitigated by acclimation (Nilsson *et al.,* 2010).

Understanding how fishes respond to warming and whether thermal acclimation can confer cross-tolerance is especially important for long-lived, late-maturing organisms that are already threatened and endangered by human impacts, such as many species of sturgeons. White Sturgeon (*Acipenser transmontanus*) are the largest and one of the longest-lived fishes in North America (Hildebrand *et al.,* 2016). Due to historic overfishing and extensive habitat alteration, many populations are threatened and endangered throughout their range (Hildebrand *et al.,* 2016). Yet despite these anthropogenic pressures, recent studies report that White Sturgeon demonstrate robust phenotypic plasticity in response to warming and low oxygen. Early juveniles from the Nechako River, British Columbia, increased both thermal and hypoxia tolerance when exposed to a 20-day heatwave, simulated from near real-time conditions being experienced in the field (Earhart *et al.,* 2023b). Furthermore, yolk-sac larvae have displayed the highest acclimation response ratio reported for fishes to date, where for every +1°C in acclimation temperature, fish increased their thermal tolerance by 1.4°C (Earhart *et al.,* 2023a).

Less is known about the potential for cross-tolerance in White Sturgeon at the southern end of their range, where they are categorized as a Species of Special Concern by the California Department of Fish and Wildlife. In the Sacramento-San Joaquin Rivers and San Francisco Estuary, White Sturgeon naturally encounter greater environmental variability (Sellheim *et al.,* 2022) compared to northern populations that spend much of their lives in relatively stable riverine habitats. Thus, it is critical to understand their capacity to respond to multiple co-occurring stressors. In this study, we aimed to characterize the thermal acclimation capacity of age-0 (young-of-the-year; YOY) White Sturgeon. To understand their capacity for thermal acclimation, fish were acclimated to five different temperatures across their natural range (14, 16, 18, 20, 22°C) for at least one month prior to measuring subsequent thermal tolerance. We predicted YOY White Sturgeon would demonstrate a high capacity for thermal acclimation, increasing thermal tolerance with increasing acclimation temperature. However, because warm acclimation may sublethally impact physiological condition, red blood cell characteristics and somatic indices were also measured to understand the health and energetic status of White Sturgeon. In addition, we aimed to understand how prior thermal exposure impacts hypoxia tolerance. We predicted acute warming would negatively impact hypoxia tolerance, but prior acclimation to warm temperatures would prompt phenotypic changes facilitating cross-tolerance to warming and hypoxia.

## Materials and Methods

### Experimental animals and acclimations

White Sturgeon were spawned May 2022 at the Sterling Caviar LLC facility (Elverta, California, USA) at 15°C and were transferred to the University of California, Davis, Center for Aquatic Biology and Aquaculture (CABA) in May 2022 following hatch. Fish were fed daily to satiation with semimoist pellets (Rangen, Inc., Buhl, Idaho) and weaned onto a dry pelleted diet (SilverCup) at ~60 days post-hatch (dph). Fish were kept at ambient temperature (18.0±0.3°C) in two large replicate 780 L tanks until ~10-month-old juveniles were moved to acclimation tanks between 13-17 March 2023.

Two circular replicate tanks per acclimation temperature were stocked with eight fish each (n = 16 per acclimation temperature). Acclimation temperatures were achieved by slowly changing from ambient temperature (18°C) by 1°C per day with a mixture of chilled, ambient, or heated well water. The temperature treatments (14, 16, 18, 20, and 22°C) encompass the preferred natural temperatures (Miller *et al.,* 2020) as well as warmer temperatures encountered in the San Francisco Estuary and Sacramento-San Joaquin Rivers, which can range from <10°C to >25°C (Wagner *et al.,* 2011; California Department of Water Resources Data Exchange Center, https://cdec.water.ca.gov/). Acclimations for the three cooler temperatures (14, 16 and 18°C) were conducted in outdoor, covered tanks filled to 235 L. Acclimations for the two warmer temperatures (20 and 22°C) were conducted in a shelter fed from the same well to maintain warmer temperatures and tanks were filled to ~190 L. Temperature was recorded daily, and dissolved oxygen (DO) and ammonia were checked periodically to ensure > 80% air saturation and < 0.25 ppm ammonia.

Upon transfer, fish were measured for mass (g) and fork length (FL; cm). Fish were size-matched and larger fish were placed in the cooler acclimation temperatures to account for differences in growth rates over the acclimation period (Table 1). One week into their acclimations, fish were anaesthetized (250 mg l^-1^ MS-222; 500 mg l^-1^ NaHCO3^-^) and tagged with Passive Integrated Transponders (PIT tags) intramuscularly on the dorsal side for tracking of individuals through experiments. Fish were fed approximately 1% body weight daily throughout the study, with the exception of fasting 72 h prior to testing. All experimental procedures in this study were conducted in accordance with the University of California Davis Institutional Animal Care and Use Committee guidelines and approved under the protocols 23064 and 23316.

**Table 1:** **Acclimation temperature and initial body condition of juvenile White Sturgeon acclimated to five different temperatures.** Upon transfer to acclimations, fish were size-matched and larger fish were placed in the cooler acclimation temperatures to account for differences in expected growth rates over the acclimation period. Temperature is represented as mean ± standard deviation, and whole-body mass (g), fork length (cm), and Fulton’s condition are represented as mean ± S.E.M. (n = 8 fish per replicate tank).

**Temperature (°C)**	**Replicate**	**Mass (g)**	**Fork length (cm)**	**Condition factor**
14.3± 0.6	1	384.4 ± 5.2	36.6 ± 0.2	0.78 ± 0.01
14.1 ± 0.6	2	383.1 ± 12.3	36.6 ± 0.5	0.78 ± 0.02
16.4 ± 0.4	1	305.0 ± 9.0	33.9 ± 0.6	0.79 ± 0.02
16.4 ± 0.5	2	352.4 ± 16.2	35.4 ± 0.4	0.79 ± 0.02
18.3 ± 0.4	1	331.9 ± 7.5	34.0 ± 0.4	0.85 ± 0.03
18.4 ± 0.4	2	322.8 ± 12.3	33.5 ± 0.4	0.86 ± 0.03
19.9 ± 0.6	1	282.6 ± 38.6	33.7 ± 0.6	0.71 ± 0.09
20.1 ± 0.8	2	290.1 ± 15.3	32.6 ± 0.6	0.84 ± 0.02
21.9 ± 1.1	1	286.4 ± 15.0	32.9 ± 1.0	0.83 ± 0.08
22.0 ± 1.1	2	307.9 ± 9.1	33.3 ± 0.6	0.84 ± 0.03

### Experimental design

**Figure 1:**
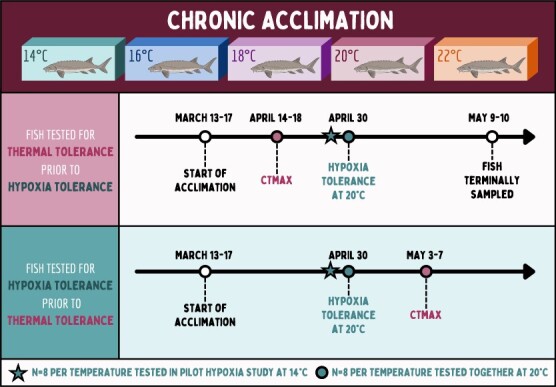
**Experimental design set-up and timeline.** White Sturgeon were acclimated to five different acclimation temperatures for one month prior to experimental tests (n = 16 per temperature). Half of the fish (n = 8 per acclimation temperature) first underwent thermal tolerance trials (critical thermal maxima; CTmax), prior to hypoxia tolerance trials. All fish underwent hypoxia trials either in a pilot study of hypoxia tolerance at 14°C (n = 8 fish per acclimation temperature; n = 4 naïve, n = 4 previously tested for thermal tolerance), or hypoxia tolerance at 20°C (n = 8 fish per acclimation temperature; n = 4 naïve, n = 4 previously tested for thermal tolerance). The second half of fish underwent thermal tolerance trials after hypoxia trials (n = 8 per acclimation temperature). Finally, fish tested for thermal tolerance prior to hypoxia tolerance were sampled for red blood cell characteristics and somatic indices (n = 8 fish per acclimation temperature).

Fish were maintained at their respective acclimation temperatures for one month prior to experimental tests starting in April 2023 (see Fig. 1 for experimental design and timeline). One month was chosen to ensure fish were demonstrating acclimatory rather than acute thermal responses (e.g., in tropical coral reef fishes, most physiological parameters stabilize between 3-5 weeks of exposure to elevated temperatures; Johansen *et al.,* 2021). The first set of critical thermal maxima (CTmax) tests was performed April 14-18, 2023, wherein eight fish from each acclimation temperature were tested for thermal tolerance, with four fish haphazardly selected from each replicate acclimation tank. A hypoxia pilot study was performed on April 29, 2023, wherein fish were directly transferred from their acclimation temperature and tested at an acute common temperature of 14°C (n = 8 fish per acclimation temperature, n = 4 per replicate tank). This pilot study was originally conducted to test if warm-acclimated White Sturgeon would completely compensate or even over-compensate for the effects of temperature on hypoxia tolerance, demonstrating higher hypoxia tolerance at 14°C. Indeed, at 14°C, no fish lost equilibrium during the ~10-h trial, demonstrating remarkable hypoxia tolerance across all acclimation temperatures but a logistically challenging experimental design. After this pilot study, a second hypoxia trial was performed on April 30, 2023, at a more challenging temperature of 20°C (representing acute warming for three of the five acclimation temperatures). For both hypoxia trials (at 14°C and 20°C), sturgeon were identified by their unique PIT tag codes, four fish were selected that had been previously tested for thermal tolerance, and four fish were selected that had not previously been tested (i.e., naïve fish). The final set of CTmax tests was performed May 3-7, 2023, on fish which had not yet been tested for thermal tolerance (n = 8 fish per acclimation temperature, n = 4 per replicate tank). Fish that underwent thermal tolerance trials prior to hypoxia tolerance trials were terminally sampled May 9-10, 2023, for red blood cell characteristics and somatic indices (n = 8 per acclimation temperature, n = 4 per replicate tank). In total, thermal tolerance was measured for all 80 fish and hypoxia tolerance at 20°C was measured for 40 of those fish, either before or after thermal tolerance trials.

### Thermal tolerance measurements

Sturgeon were randomly selected for the initial thermal tolerance trials and tested at their respective acclimation temperatures to establish CTmax (originally developed by Cowles and Bogert, 1944, and recently reviewed by Desforges *et al.,* 2023), in a method similar to Beitinger *et al.* (2000). Eight fish were measured per acclimation temperature and split into one of two trials per day. Four fish were tested per trial, either the late morning or early afternoon. Each individual was placed into individual half-filled 37 L glass aquarium tanks (18.5 L water volume), situated within a water table connected to an external heating system. Fish were allowed to adjust for 30 min prior to the start of each trial. To maintain oxygen levels, each tank was aerated, and DO was measured using a YSI 556 Multiparameter System at the beginning and end of each trial for each tank (across all trials, starting DO: 95.66 ± 0.37% air saturation; final DO: 82.72 ± 0.54% air saturation). Reduction from 100% to 80% DO did not impact CTmax for juvenile white sturgeon (unpublished data; M. Bédard) and was not expected impact CTmax data here. Following the 30-min adjustment period, temperature was increased 0.3°C min^-1^. Individual tank temperatures were recorded each minute using a Thermapen® ONE thermometer (ThermoWorks©; NIST calibration certified, 0.1°C precision). CTmax was recorded as the temperature (°C) when loss of equilibrium (LOE) occurred. LOE was determined when the fish could no longer maintain upright orientation (i.e., rolling over and exposing their belly for more than 5 s). Immediately upon LOE, fish were removed from their test tank, measured for mass and FL, then placed in a recovery tank at +6°C acclimation temperature to prevent cold shock post-CTmax. After a 2-h recovery, fish were placed back in their respective acclimation tanks. There were no mortalities throughout the experiment of fish that underwent CTmax prior to hypoxia tolerance testing.

After hypoxia tolerance trials (described below), followed by at least a 72-h recovery period, sturgeon were identified by their unique PIT tag codes and fish that had not previously undergone CTmax trials were tested for CTmax in the same manner as above (n = 8 per acclimation temperature). There was one mortality of a 22°C-acclimated fish which underwent hypoxia tolerance testing prior to CTmax, occurring two days post-CTmax. We used CTmax data to determine thermal plasticity with warm acclimation, by calculating the overall acclimation response ratio (ARR) between 14°C and 22°C, as well as the ARR between each sequential increase in acclimation temperature:


$$ ARR=\left(\frac{CT_{\mathit{\max}2}-{CT}_{\mathit{\max}1}}{T_2-{T}_1}\right) $$

where CTmax2 and CTmax1 are the mean critical thermal maxima for different acclimation groups and *T_2_* and *T_1_* are the respective acclimation temperatures.

### Hypoxia tolerance measurements

Hypoxia tolerance at 20°C was measured using a modified hypoxia challenge test as described in Claireaux *et al.* (2013) and Strowbridge *et al.* (2021). Briefly, fish were transferred from their acclimation tanks (n = 8 per acclimation temperature) to an aerated ~600 L tank. One replicate tank per acclimation temperature was used in this experiment, ensuring an even mix of fish previously tested for CTmax (n = 4 per acclimation temperature) and naïve fish (n = 4 per acclimation temperature). The test tank was maintained at 20°C with a titanium aquarium heater. After a 15-min adjustment period, DO was lowered by bubbling nitrogen into the test tank, which was split evenly through four micro-bubble air stones and one air line placed in the outflow of a small circulation pump. This was accompanied by a second small circulation pump to ensure consistent mixing of nitrogen throughout the tank. Temperature (°C) and DO (% air saturation) were monitored in real-time using a YSI 556 Multiparameter System and Loligo© Systems Witrox oxygen sensor, fiber optic oxygen and temperature probes, in conjunction with AquaResp, which reported temperature-compensated oxygen values in real-time. DO was decreased ~1% air saturation min^-1^ until 20% air saturation was reached, at which point the rate of decrease was lowered to ~2% air saturation h^-1^ to increase the resolution of ILOS measurements as per Claireaux *et al.* (2013).

Incipient lethal oxygen saturation (ILOS) was determined as the DO (% air saturation) at which individuals demonstrated LOE. Immediately upon LOE, fish were identified by their unique PIT tag codes and removed from the test tank into a well-aerated recovery tank at 20°C for at least 30 min prior to returning to their original acclimation tanks. However, DO did not drop below 6% air saturation with nitrogen bubbling due to air mixing and fish movement, and individuals that remained at 6% air saturation demonstrated changes in behavior to minimize gill ventilation and activity. Due to this inability to decrease DO further and the stamina of some individuals to withstand low oxygen, the hypoxia trial was ended by the experimenter (AMD) after 905 min (~15h) of hypoxia exposure. “Hypoxia exposure” is considered as the period after the rate of decrease was lowered to ~2% air saturation h^-1^ (average DO: 12.03 ± 0.01 % air saturation). Thus, hypoxia tolerance is additionally assessed as time in hypoxia (min). The individuals that outlasted the experimenter were then assigned an arbitrary hypoxia tolerance value of 0% air saturation and 905 min. After hypoxia tolerance trials, fish that underwent CTmax prior to hypoxia tolerance testing were recovered for at least one week to mitigate any transient changes from acute hypoxia exposure prior to terminal sampling.

### Somatic indices and red blood cell characteristics

Fish that underwent CTmax prior to hypoxia tolerance testing (n = 8 per acclimation temperature) were euthanized (500 mg l^-1^ MS-222; 1 g l^-1^ NaHCO3^-^), then measured for fork length (FL; measured to the nearest 0.5 cm) and whole-body mass (measured to the nearest 1 g). Fulton’s condition factor (Fulton, 1904; Nash *et al.,* 2006) was calculated as follows:


$$ Condition\ factor=\frac{Whole\ body\ mass\ (g)\times 100}{Fork\ length\ {(cm)}^3} $$

Whole blood and tissue samples were collected and processed as described in Weber *et al.* (2024). Blood was sampled from the caudal vein using a heparinized needle and syringe for hematocrit (Hct; %) and hemoglobin concentration (Hb; mM) measurements. Whole blood was collected into micro-capillary tubes in triplicate per individual and centrifuged for 2 min at 10,000 rpm. The ratio of packed red blood cells volume to whole blood volume was then calculated and averaged per individual for Hct. For Hb, 4 μl of whole blood was diluted in 1 ml Drabkin’s solution, and samples were kept at 4°C overnight prior to measurement. Each sample was then measured in triplicate at 540 nm. Average Hb was calculated using absorbance (A) and an extinction coefficient of 11 mmol l^-1^ cm^-1^, and accounting for a 1:250 dilution factor and 4 Heme groups:


$$ HB=\frac{250\left(A/11\right)}{4} $$

Mean Corpuscular Hemoglobin Concentration (MCHC) was calculated by dividing Hb concentration by Hct:


$$ MCHC= Hb/\left(\frac{Hct}{100}\right) $$

Liver, spleen, and the ventricle were dissected from the fish and their respective mass was measured to the nearest 1 g. Hepatosomatic Index (HSI) and Splenic Somatic Index (SSI) were calculated by dividing liver or spleen mass by whole-body mass and multiplying by 100. Relative ventricular mass (RVM) was calculated by converting ventricular mass (g) to mg and dividing by whole-body mass.

### Statistical analyses

All statistical analyses were performed using RStudio (version 2022.12.0; http://www.R-project.org/) with a significance level (α) of 0.05. All data were tested for normality using Shapiro-Wilk’s tests and homogeneity of variance using Levene’s tests prior to analyses. Where data failed normality, as was the case for HSI, a log transformation was used. To assess the effect of acclimation temperature on each measurement, a series of one-way ANOVAs were performed using the packages *afex* (Singmann *et al.,* 2015) and *emmeans* (Lenth *et al.,* 2018). No tank effects (differences in replicate acclimation tanks) were observed on any measurements. Fish IDs (individuals identified by their unique PIT tag codes) were also included as a random effect. Multiplicative two-way ANOVAs were also used to assess if experiment order (if fish underwent CTmax then ILOS, or ILOS then CTmax) had a main effect on thermal tolerance and hypoxia tolerance, and if there was a significant interaction with acclimation temperature (i.e., assuming the effect of experiment order may be different for each acclimation temperature). If acclimation temperature or experiment order had a significant effect on a measurement, Tukey HSD post-hoc tests were conducted, and results are reported with Tukey adjusted p-values. Detailed outputs of Tukey HSD post-hoc tests can be found in Supplementary Table S1-3.

Furthermore, we calculated Cohen’s *d* and corresponding 95% confidence intervals (CI; generated through bias-corrected and accelerated bootstrap resampling) using the package *dabestr* (Ho *et al.,* 2019) for somatic indices, where p-values were > 0.05 but trends in data suggested physiological significance. These effect sizes were calculated as compared to the control acclimation temperature of 14°C. Effect size is relatively small if *d* = 0.2, medium if *d* = 0.5, and large (i.e., biologically meaningful) if *d* = 0.8 (Sullivan and Feinn, 2012; Cohen, 2013). All data are reported as mean ± S.E.M.

## Results

### Thermal tolerance

**Figure 2:**
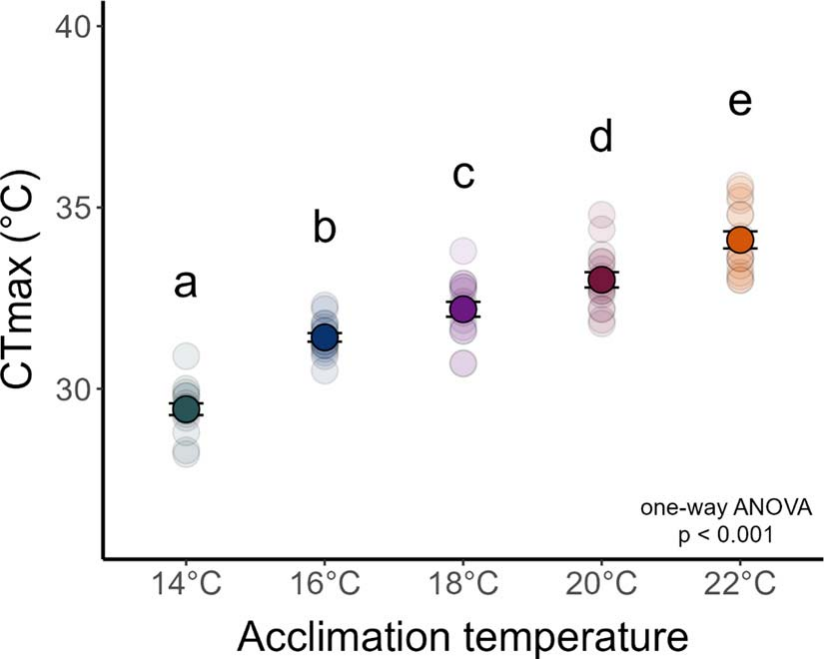
**Critical thermal maxima (CT**_**max**_ **) of juvenile White Sturgeon acclimated to five different temperatures.** Letters denote statistically significant differences as detected by Tukey HSD post-hoc analysis (see Supplementary Table S1 for details). Individual data points (light circles; n = 16 per acclimation temperature) are overlayed with mean ± S.E.M. (dark circles ± error bars).

There was a significant effect of acclimation temperature on CTmax (Fig. 2; F4,75 = 84.81, p < 0.001). Post-hoc analyses (Supplementary Table S1) showed significant increases in CTmax with each +2°C increase in acclimation temperature: 14°C (CTmax = 29.4 ± 0.2), 16°C (CTmax = 31.4 ± 0.1), 18°C (CTmax = 32.2 ± 0.2), 20°C (CTmax = 33.0 ± 0.2), and 22°C (CTmax = 34.1 ± 0.2). Across all 80 CTmax trials, average ramp rate was 0.29 ± 0.002 °C min^-1^ but varied from 0.25-0.33 °C min^-1^. However, we found that ramp rate was not a significant predictor of CTmax (Pearson’s correlation; R = -0.11, p = 0.32). There was no main effect of experiment order (two-way ANOVA; F1,70 = 1.64, p = 0.204) nor an interaction with acclimation temperature (F4,70 = 1.55, p = 0.198). We found a significant positive linear relationship between CTmax across all temperature acclimations (Supplementary Fig. S1; p < 0.001, adjusted R^2^ = 0.79, slope = 0.55). The overall ARR between 14-22°C was 0.58. ARR was highest between 14-16°C (0.99) and lowest between 16-18°C (0.39). ARR between 18-20°C was 0.41, and ARR between 20-22°C was 0.55.

### Hypoxia tolerance

**Figure 3:**
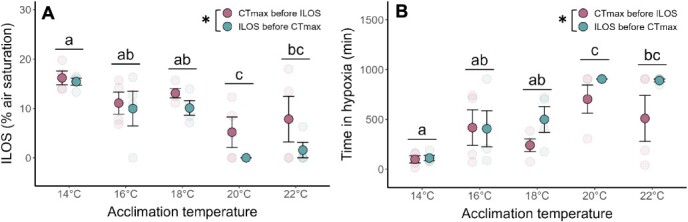
**Hypoxia tolerance of juvenile White Sturgeon acclimated to five different temperatures and measured at 20°C for** (**A**) **incipient lethal oxygen saturation (ILOS; % air saturation) and (B) time in hypoxia (in minutes).** There were significant main effects of experiment order (whether fish underwent CTmax trials before ILOS trials, or vice versa) and acclimation temperature (see Results for details). Asterisks denote statistically significant differences between experiment order, and lowercase letters denote statistically significant differences between acclimation temperatures as detected by Tukey HSD post-hoc analysis (see Supplementary Table S2 for details). Individual data points (light circles; n = 4 per experiment order per acclimation temperature) are overlayed with mean ± S.E.M. (dark circles ± error bars).

There was a main effect of acclimation temperature on ILOS (Fig. 3A; two-way ANOVA; F4,30 = 10.14, p < 0.001) and main effect of experiment order (F1,30 = 4.71, p = 0.038) but no interaction between acclimation temperature and experiment order (F4,30 = 0.53, p = 0.718). In addition, there was a main effect of acclimation temperature on time in hypoxia (Fig. 3B; two- way ANOVA; F4,30 = 9.67, p < 0.001) and a main effect of experiment order (F1,30 = 4.43, p = 0.044) but no interaction between acclimation temperature and experiment order (F4,30 = 0.86, p = 0.497). Post-hoc analyses (Supplementary Table S2) showed significantly higher hypoxia tolerance (decreased ILOS and increased time in hypoxia) for fish acclimated to warmer temperatures, where 20°C-acclimated fish had the greatest hypoxia tolerance and 14°C-acclimated fish had the lowest hypoxia tolerance. In addition, naïve fish demonstrated significantly higher hypoxia tolerance than fish that had previously been tested for thermal tolerance. Supplementary Table S4 provides raw data of hypoxia tolerance for each experiment order and acclimation temperature.

### Somatic indices and red blood cell characteristics

There was no significant effect of acclimation temperature on body condition (Table 2): mass (F4,35 = 0.79, p = 0.543), fork length (F4,35 = 1.36, p = 0.267), and condition factor (F4,35 = 1.68, p = 0.176). Also, there was no significant effect of acclimation temperature on hematological characteristics (Table 3): hematocrit (F4,34 = 0.79, p = 0.541), hemoglobin (F4,35 = 0.89, p = 0.482), and MCHC (F4,34 = 1.04, p = 0.402).

**Table 2:** **Final body condition of juvenile White Sturgeon acclimated to five different temperatures.** At the end of the experiment, whole-body mass (g), fork length (cm), and Fulton’s condition factor were measured for all fish that underwent thermal tolerance tests prior to hypoxia tolerance. There were no significant effects of acclimation temperature on body condition (one-way ANOVA, p > 0.05; see Results for details). All data is represented as mean ± S.E.M. (n = 8 fish per acclimation temperature).

**Acclimation temperature**	**Mass (g)**	**Fork length (cm)**	**Condition factor**
14°C	474.26 ± 24.55	40.6 ± 0.9	0.71 ± 0.03
16°C	441.75 ± 31.11	39.4 ± 0.8	0.72 ± 0.02
18°C	427.84 ± 18.24	38.3 ± 0.4	0.76 ± 0.03
20°C	427.05 ± 21.74	38.7 ± 1.0	0.74 ± 0.02
22°C	419.74 ± 25.04	39.4 ± 0.6	0.68 ± 0.02

**Table 3:** **Red blood cell characteristics and somatic indices of juvenile White Sturgeon acclimated to five different temperatures**. There were no significant effects of acclimation temperature on hematocrit (Hct), hemoglobin (Hb), mean corpuscular hemoglobin concentration (MCHC), hepatosomatic index (HSI), and splenic somatic index (SSI) (one-way ANOVA, p > 0.05; see Results for details). There was a significant effect of acclimation temperature (*) on relative ventricular mass (RVM). Letters denote statistically significant differences between RVM based on the subsequent Tukey HSD post-hoc analyses. All data is represented as mean ± S.E.M. (n = 8 fish per acclimation temperature, except n = 7 for hematocrit and MCHC at 20°C).

**Acclimation temperature**	**Hct (%)**	**Hb [mM]**	**MCHC**	**HSI**	**SSI**	RVM*
14°C	21.56 ± 1.36	1.01 ± 0.06	4.70 ± 0.23	2.045 ± 0.248	0.249 ± 0.024	1.138 ± 0.045^a^
16°C	19.84 ± 1.78	0.94 ± 0.05	4.89 ± 0.27	1.710 ± 0.155	0.242 ± 0.024	1.125 ± 0.048^ab^
18°C	21.16 ± 0.84	0.92 ± 0.04	4.40 ± 0.26	1.464 ± 0.112	0.243 ± 0.026	0.922 ± 0.049^bc^
20°C	22.84 ± 0.71	1.00 ± 0.04	4.38 ± 0.21	1.489 ± 0.105	0.207 ± 0.015	0.922 ± 0.065^bc^
22°C	20.85 ± 0.88	0.91 ± 0.03	4.40 ± 0.12	1.467 ± 0.099	0.188 ± 0.007	0.913 ± 0.046^c^

**Figure 4:**
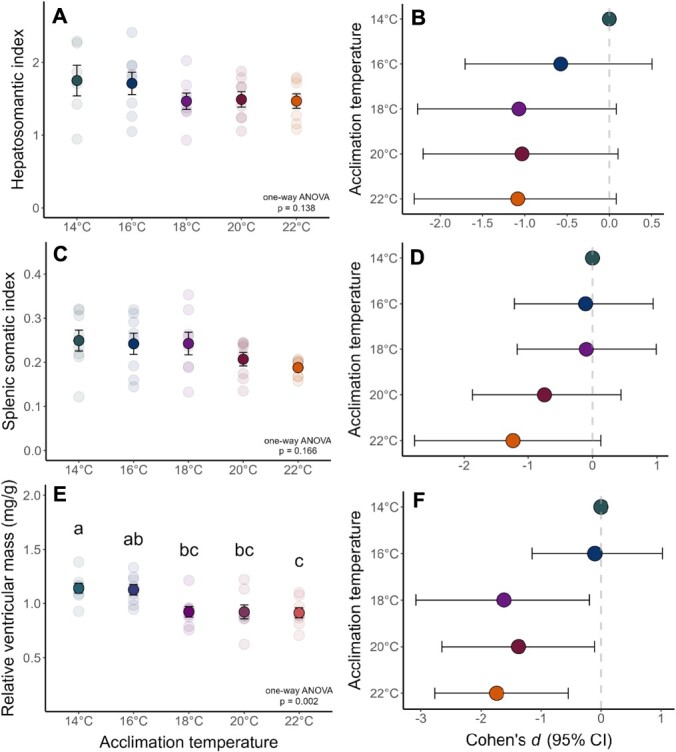
**Somatic indices of juvenile White Sturgeon acclimated to five different temperatures.** Somatic indices and Cohen’s *d* values (with 95% confidence intervals) were calculated for hepatosomatic index (**A-B**), splenic somatic index (**C-D**), and relative ventricular mass (**E-F**). Letters denote statistically significant differences between acclimation temperatures for relative ventricular mass (**E**) as detected by Tukey HSD post-hoc analysis (see Supplementary Table S3 for details). Cohen’s *d* values demonstrate the magnitude of effect for each acclimation temperature as compared to the control temperature of 14°C (where effect size is small if *d* = 0.2, medium if *d* 731 = 0.5, and large if *d* = 0.8). For somatic indices, individual data points (light circles; n = 8 per acclimation temperature) are overlaid with mean ± S.E.M. (dark circles ± error bars).

There was no significant effect of acclimation temperature on HSI (Table 3; Fig. 4A; F4,35 = 1.87, p = 0.138). For 16°C-, 18°C-, 20°C-, and 22°C-acclimated fish, the effect size of acclimation temperature on HSI was -0.575, -1.069, -1.033, and -1.083, respectively (Fig. 4B). There was no significant effect of acclimation temperature on SSI (Table 3; Fig. 4C; F4,35 = 1.73, p = 0.166). For 16°C-, 18°C-, 20°C-, and 22°C-acclimated fish, the effect size of acclimation temperature on SSI was -0.107, -0.095, -0.746, and -1.233, respectively (Fig. 4D). There was a significant effect of acclimation temperature on RVM (Table 3; Fig. 4E; F4,35 = 5.19, p = 0.002). Post-hoc analyses (Supplementary Table S3) showed significant decreases in RVM with warmer acclimation temperature. For 16°C-, 18°C-, 20°C-, and 22°C-acclimated fish, the effect size of acclimation temperature on RVM was -0.101, -1.617, -1.373, and -1.738, respectively (Fig. 4F).

## Discussion

The main objectives of this study were to characterize the thermal acclimation capacity of southern White Sturgeon, and to understand the potential for cross-tolerance by identifying how prior thermal exposure impacts their ability to tolerate low oxygen. As expected, YOY White Sturgeon increased their upper thermal limit and demonstrated greater hypoxia tolerance with acclimation to warmer temperatures. Warm-acclimated Sturgeon also had lower relative ventricular mass, while other somatic indices, red blood cell characteristics, body conditions were not affected. Most strikingly, even with at least one week of recovery, prior exposure to acute thermal stress (CTmax) reduced the fish’s tolerance to hypoxia compared to naïve fish. Therefore, prior exposure to warming, and whether that exposure is acute or chronic, are important considerations for understanding White Sturgeon phenotypic plasticity and responses to changing environments.

In comparison to other ectothermic species, YOY White Sturgeon demonstrated a high capacity for acclimation (Gunderson and Stillman, 2015; Morley *et al.,* 2019). Though aquatic and marine ectotherms have notably higher ARRs than terrestrial ectotherms, previously reported values are generally < 0.4 (Gunderson and Stillman, 2015; Morley *et al.,* 2019). Here, YOY White Sturgeon demonstrated the capacity to increase their upper thermal limit by 0.58°C per 1°C of acclimation temperature in response to a +8°C acclimation. This is comparable to the ARR of older subadult White Sturgeon from the Fraser River, British Columbia, which increase their upper thermal limit 0.52°C per 1°C of acclimation temperature in response to +6°C (Weber *et al.,* 2024). Interestingly, we found this increase in upper thermal limit to be dependent on which acclimation temperatures were used for comparisons. For example, the positive linear relationship between CTmax and all acclimation temperatures indicates overall, White Sturgeon increase upper thermal limit 0.55°C per 1°C of acclimation temperature. Between acclimation temperatures, fish demonstrated the highest acclimation capacity between 14 and 16°C with an ARR of 0.99. As aforementioned, yolk-sac larvae White Sturgeon recently demonstrated one of the highest ARRs reported thus far (1.4) when acclimated to 14 and 18°C (Earhart *et al.,* 2023a). This ARR decreased to 0.9 between 18 and 21°C (Earhart *et al.,* 2023a), and it is thought that for northern species of sturgeon, plasticity decreases as acclimation temperatures approach 20°C (Zhang and Kieffer, 2014; Bugg *et al.,* 2020; Bugg *et al.,* 2023; Penman *et al.,* 2023). While we do not see a plateauing effect with upper thermal limits in the YOY southern White Sturgeon examined here, ARR is lower between subsequent acclimation temperatures and thermal safety margins (the difference between upper thermal limit and acclimation temperature) decrease from 15.4°C for 14°C-acclimated fish to 12.1°C for 22°C-acclimated fish. This suggests that YOY White Sturgeon have a great capacity for acclimation and a wide thermal safety margin to buffer from changing temperature, but that these advantageous characteristics decline with warmer acclimation temperatures.

Perhaps unsurprisingly, we found that fish acclimated to warm temperatures demonstrated the best hypoxia tolerance at 20°C, similar to other fish species (Anttila *et al.,* 2015; McBryan *et al.,* 2016). For example, acute warming decreased hypoxia tolerance in *Fundulus heteroclitus*, but with warm acclimation, fish partially compensated for the acute thermal stress and improved hypoxia tolerance (McBryan *et al.,* 2016). We predicted that warm-acclimated YOY White Sturgeon would completely compensate or even over-compensate for the effects of temperature on hypoxia tolerance, demonstrating higher hypoxia tolerance at 14°C. Unfortunately, the pilot study was unsuccessful in measuring hypoxia tolerance at 14°C, as all fish surprisingly did not demonstrate loss of equilibrium within ~10 h. While follow-up studies are needed to properly quantify these predicted compensatory responses, Earhart *et al.* (2023b) demonstrated that early life stage White Sturgeon exposed to prolonged warming completely compensated for acute thermal stress during hypoxia tolerance trials. Acute thermal exposure at 17°C and 20°C impaired hypoxia tolerance for fish acclimated to 13°C, but fish acclimated to and tested at 20°C demonstrated nearly identical hypoxia tolerance to 13°C-acclimated fish tested at 13°C (Earhart *et al.,* 2023b).

While White Sturgeon did not demonstrate any changes in red blood cell characteristics between acclimation temperatures, we found a pattern of decreasing relative tissue indices with warmer temperatures. Interestingly, acclimation temperature did not have a significant effect on HSI or SSI. Though effect sizes of temperature were large for warm-acclimated fish, the confidence intervals calculated with Cohen’s *d* suggest that this declining pattern is not significant for HSI or SSI. This is inconsistent with previous studies of sturgeon and other fishes, where HSI significantly decreases with warmer temperature and may indicate increased mobilization of energy stores with increased energetic costs (Rossi *et al.,* 2017; Bugg *et al.,* 2020; Penman *et al.,* 2023). Instead, warm-acclimated White Sturgeon demonstrated significantly lower RVM. Previous thermal acclimation studies have also shown a reduction in RVM in warm-acclimated fish (Anttila *et al.,* 2015; Nyboer and Chapman, 2018) and larger RVM in cold-acclimated fish (Klaiman *et al.,* 2011), caused by structural remodeling of the heart during thermal acclimation. While warming increases oxygen demand, fish can develop thicker, stronger compact myocardium to generate more pressure and increase or maintain cardiac output, which is correlated with decreased RVM (Anttila *et al*., 2015; Klaiman *et al.,* 2011; Nyboer and Chapman, 2018). In contrast, RVM is positively correlated with CTmax in Atlantic salmon, where larger RVM is thought to be associated with increased tissue oxygen delivery to match the elevated oxygen demand of acute thermal stress (Anttila *et al.,* 2013). Warm-acclimated White Sturgeon demonstrated both high CTmax and lower RVM, which may imply that for White Sturgeon, upper thermal limits are not constrained by oxygen transport mechanisms. However, more studies are needed to understand the compensatory responses of the White Sturgeon cardiac system in response to acute and prolonged warm temperatures.

Notably, prior CTmax testing affected the White Sturgeon response to hypoxia, which may have implications for both future experimental design considerations and real-world scenarios. This pattern has previously been seen in rainbow trout (*Oncorhynchus mykiss*) wherein previous hypoxia exposure did not impact thermal tolerance, but hypoxia tolerance decreased with previous CTmax exposure (Strowbridge *et al.,* 2021). We found that this effect of prior acute thermal stress on hypoxia tolerance depended on acclimation state, as warm-acclimated fish were affected more severely than fish acclimated to 14°C and 16°C (Fig. 3). This may suggest that cool-acclimated fish are able to recover from the physiological challenge of CTmax faster than warm-acclimated fish, or that warm-acclimated fish, which reach greater temperatures during CTmax trials, are incurring greater thermal stress. Furthermore, Strowbridge *et al.* (2021) found that when fish underwent hypoxia trials first, post-trial mortality significantly increased post-CTmax, despite 2-3 weeks of recovery between experiments. Similarly, we found one delayed mortality post-CTmax for an individual that had been tested for hypoxia tolerance; though notably, this fish was acclimated to the warmest temperature (22°C) and underwent a short recovery time before CTmax. These post-CTmax mortalities suggest that acute low oxygen stress may incur greater energetic costs or necessitate a longer recovery period than acute warming in a way that interacts negatively with subsequent acute warming stress. With both natural and climate change-induced fluctuations of oxygen and temperature, there is a need for quantitative assessments of post-stress recovery to better characterize White Sturgeon vulnerability to warming and hypoxia.

It is also of note that the hypoxic conditions of the tolerance trial reported here are more extreme than the daily average dissolved oxygen levels of the Delta monitored by the California’s Department of Water Resources (at or above 5 mg/L DO). We found the least hypoxia tolerant group of fish were acclimated to 14°C and experienced the most extreme change in temperature during this test (+6°C); yet, these fish persisted in hypoxia for nearly two hours (106 min) and lost equilibrium at 15.79% air saturation, or roughly 1.4 mg/L DO, on average. During the hypoxia tolerance trial, fish were observed to have decreased activity and ventilation frequency, which may indicate whole-animal mechanisms by which these fish are able to compensate for hypoxia and thermal stress (Farrell and Richards, 2009; Blewett *et al.,* 2022; Earhart *et al.,* 2022). Though these were opportune, qualitative observations, this highlights the need to quantitatively assess how behavior modulates White Sturgeon stress tolerance. Geist *et al.* (2005) found White Sturgeon demonstrate temperature-dependent oxygen consumption, increasing metabolism with increased temperature, but little is known about how their metabolism changes in response to both hypoxia and warming. Perhaps, like hypoxia-tolerant species, White Sturgeon more readily utilize anaerobic mechanisms (Richards, 2009). In addition, their extraordinary capacity for tolerating acid-base disturbances (Baker and Brauner, 2012; Shartau *et al.,* 2017) could play a role in their hypoxia tolerance. Though more work is needed, our data suggest that White Sturgeon have an innate capacity to tolerate stressful conditions that exceed their currently experienced natural conditions.

However, global climate change is inducing more frequent and intense shifts in environmental conditions, often with cascading effects. Recently, the San Francisco Estuary experienced unprecedented White Sturgeon mortality events during the warm summer months of 2022 and 2023, coinciding with red tide algal blooms (*Heterosigma akashiwo*). These blooms are well-known to cause fish mortality, reducing ambient oxygen levels and producing lethal toxins even at oxygen levels > 15.3 mg/L DO (Black *et al.,* 1991). *H. akashiwo* populations that sink to depths are significantly more toxic (Powers *et al.,* 2012), which would particularly endanger benthic species like White Sturgeon. Importantly, these mortality events have prompted reconsideration of the species’ status with the U.S. Endangered Species Act, to amend this status from unlisted to “threatened”. Further study is needed to characterize the physiological responses of White Sturgeon to thermal stress in low oxygen scenarios, but with the critical addition of co-occurring toxins, like those present in *H. akashiwo* blooms, as well as other contaminants affecting White Sturgeon in the San Francisco Estuary (Gundersen *et al*., 2017). Understanding what underlies tolerance and plasticity in response to these multiple stressors is essential for predicting and enhancing the long-term success of southern White Sturgeon.

## Supplementary Material

Web_Material_coae089

## Data Availability

Data used in this study are archived in the repository figshare (https://doi.org/10.6084/m9.figshare.25360378.v1).
