# Supplementary material for: Prior thermal acclimation gives White Sturgeon a fin up dealing with low oxygen
Source: Conserv Physiol. 2025 Jan 6;13(1):coae089. doi: 10.1093/conphys/coae089 (PMC11704418; doi:10.1093/conphys/coae089)
Supplement: Web_Material_coae089 [file web_material_coae089.zip › Graphical abstract.pdf]

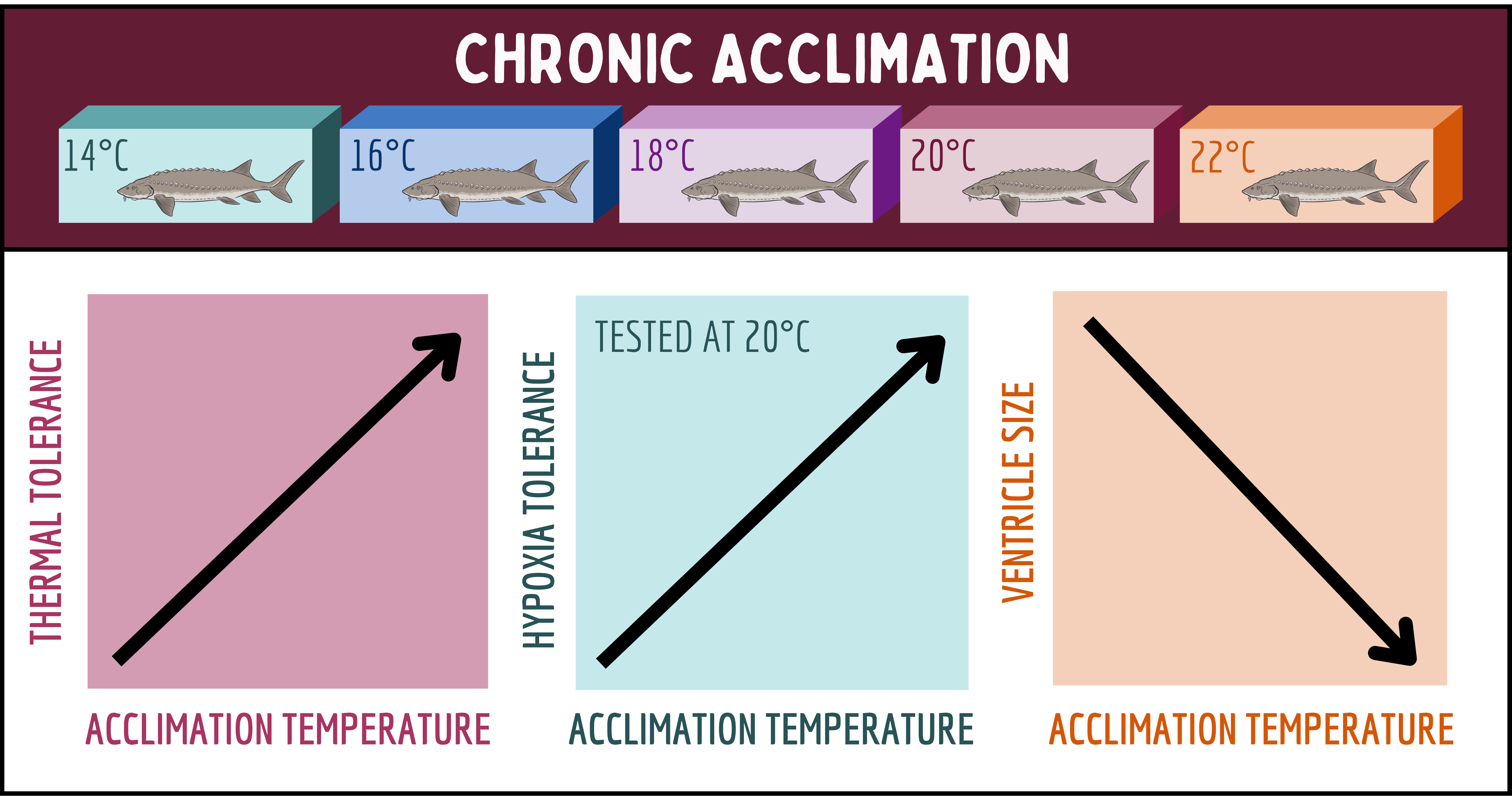
CHRONIC ACCLIMATION
14°C
16°C
18°C
20°C
22°C
THERMAL TOLERANCE
ACCLIMATION TEMPERATURE
HYPOXIA TOLERANCE
TESTED AT 20°C
ACCLIMATION TEMPERATURE
VENTRICLE SIZE
ACCLIMATION TEMPERATURE
