# Supplementary material for: Prior thermal acclimation gives White Sturgeon a fin up dealing with low oxygen
Source: Conserv Physiol. 2025 Jan 6;13(1):coae089. doi: 10.1093/conphys/coae089 (PMC11704418; doi:10.1093/conphys/coae089)
Supplement: Web_Material_coae089 [file web_material_coae089.zip › Supplementary Materials.pdf]

Supplementary Materials

Title: Prior thermal acclimation gives White Sturgeon a fin up dealing with low oxygen

Authors: Angelina M. Dichiera[1,2*], Kelly D. Hannan[3], Garfield T. Kwan[3], Nann A. Fangue[3], Patricia M. Schulte[2], Colin J. Brauner[2]

Affiliations:

[1] Virginia Institute of Marine Science, William & Mary, Gloucester Point, Virginia, USA (present address)

[2] Department of Zoology, The University of British Columbia, Vancouver, British Columbia, Canada

[3] Department of Wildlife, Fish and Conservation Biology, University of California Davis, Davis, California, USA

*Correspondence to: dichiera@vims.edu

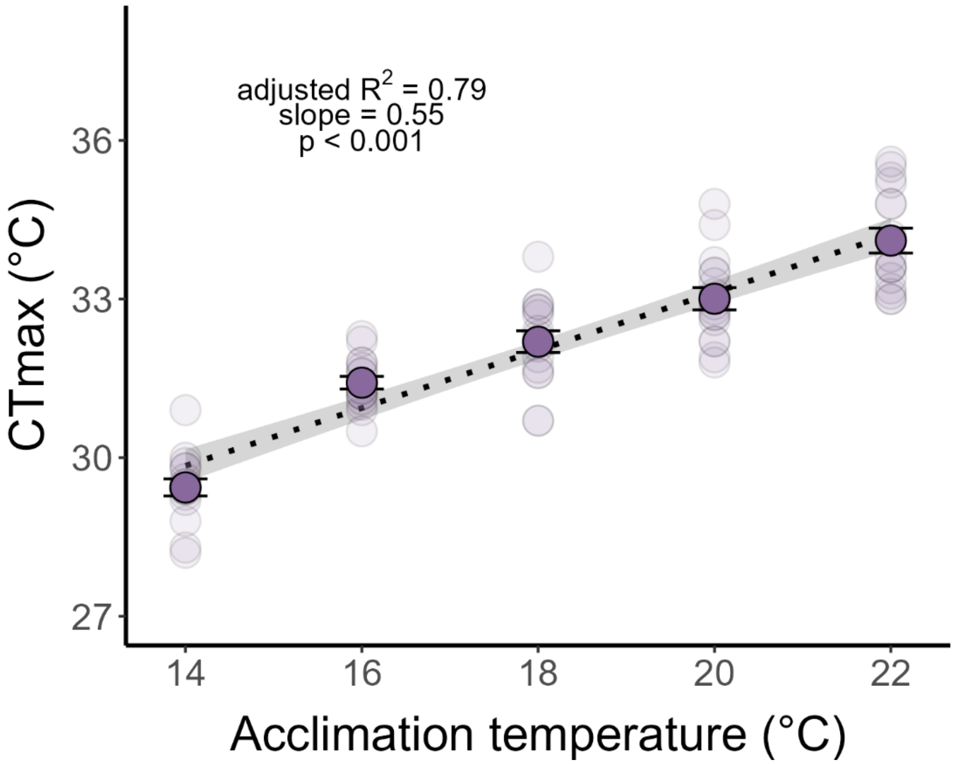

Figure S1. Linear relationship between critical thermal maxima (CT_{max}) of juvenile White Sturgeon and acclimation temperature. Dashed line and surrounding grey area indicate linear regression and 95% confidence interval. Individual data points (light circles; n = 16 per acclimation temperature) are overlayed with mean ± S.E.M. (dark circles ± error bars).

Table S1. Thermal tolerance Tukey HSD post-hoc tests for multiple comparisons. After significant effects of acclimation temperature on thermal tolerance (CT_{max}; critical thermal maxima) were found, pairwise comparisons were used to identify significant differences between acclimation temperatures. P-values (adjusted by the Tukey method) in bold denote statistical significance where significance level (α) = 0.05.

Metric	Comparison	estimate	SE	df	t ratio	p-value
**CT_{max}**	14°C vs 16°C	-1.981	0.27	75	-7.332	**<0.0001**
	14°C vs 18°C	-2.756	0.27	75	-10.200	**<0.0001**
	14°C vs 20°C	-3.569	0.27	75	-13.207	**<0.0001**
	14°C vs 22°C	-4.669	0.27	75	-17.278	**<0.0001**
	16°C vs 18°C	-0.775	0.27	75	-2.868	**0.0415**
	16°C vs 20°C	-1.587	0.27	75	-5.875	**<0.0001**
	16°C vs 22°C	-2.688	0.27	75	-9.946	**<0.0001**
	18°C vs 20°C	-0.812	0.27	75	-3.007	**0.0287**
	18°C vs 22°C	-1.913	0.27	75	-7.078	**<0.0001**
	20°C vs 22°C	-1.100	0.27	75	-4.071	**0.0011**

Table S2. Hypoxia tolerance Tukey HSD post-hoc tests for multiple comparisons. After significant main effects of experiment order and acclimation temperature on incipient lethal oxygen saturation (ILOS) and time in hypoxia were found, pairwise comparisons were used to identify significant differences between experiment order and significant differences between acclimation temperatures. P-values (adjusted by the Tukey method) in bold denote statistical significance where significance level (α) = 0.05.

Metric	Comparison	estimate	SE	df	t ratio	p-value
**ILOS**	CT_{max} first vs ILOS first	3.26	1.5	30	2.170	**0.0380**
	14°C vs 16°C	5.26	2.38	30	2.211	0.2031
	14°C vs 18°C	4.20	2.38	30	1.765	0.4120
	14°C vs 20°C	13.20	2.38	30	5.550	**<0.0001**
	14°C vs 22°C	11.08	2.38	30	4.659	**0.0005**
	16°C vs 18°C	-1.08	2.38	30	-0.447	0.9913
	16°C vs 20°C	7.94	2.38	30	3.339	**0.0178**
	16°C vs 22°C	5.82	2.38	30	2.448	0.1303
	18°C vs 20°C	9.00	2.38	30	3.786	**0.0057**
	18°C vs 22°C	6.88	2.38	30	2.895	0.0506
	20°C vs 22°C	-2.12	2.38	30	-0.891	0.8981
**Time in hypoxia**	CT_{max} first vs ILOS first	-168	80.1	30	-2.104	**0.0439**
	14°C vs 16°C	-305.9	127	30	-2.415	0.1389
	14°C vs 18°C	-264.4	127	30	-2.088	0.2516
	14°C vs 20°C	-698.1	127	30	-5.513	**0.0001**
	14°C vs 22°C	-594.1	127	30	-4.692	**0.0005**
	16°C vs 18°C	41.5	127	30	0.328	0.9974
	16°C vs 20°C	-392.2	127	30	-3.097	**0.0318**
	16°C vs 22°C	-288.2	127	30	-2.276	0.1806
	18°C vs 20°C	-433.8	127	30	-3.425	**0.0144**
	18°C vs 22°C	-329.8	127	30	-2.604	0.0951
	20°C vs 22°C	104.0	127	30	0.821	0.9220

Table S3. Relative ventricular mass Tukey HSD post-hoc tests for multiple comparisons. After significant effects of acclimation temperatures on relative ventricular mass (RVM) were found, pairwise comparisons were used to identify significant differences between acclimation temperatures. P-values (adjusted by the Tukey method) in bold denote statistical significance where significance level (α) = 0.05.

Metric	Comparison	estimate	SE	df	t ratio	p-value
**RVM**	14°C vs 16°C	0.0134	0.0724	35	0.185	0.9997
	14°C vs 18°C	0.2161	0.0724	35	2.984	**0.0387**
	14°C vs 20°C	0.2167	0.0724	35	2.993	**0.0379**
	14°C vs 22°C	0.2253	0.0724	35	3.112	**0.0284**
	16°C vs 18°C	0.2026	0.0724	35	2.798	0.0596
	16°C vs 20°C	0.2033	0.0724	35	2.808	0.0583
	16°C vs 22°C	0.2119	0.0724	35	2.926	**0.0444**
	18°C vs 20°C	0.007	0.0724	35	0.010	1.0000
	18°C vs 22°C	0.0093	0.0724	35	0.128	0.9999
	20°C vs 22°C	0.0086	0.0724	35	0.118	1.0000

Table S4. Hypoxia tolerance of juvenile White Sturgeon acclimated to five different temperatures (measured at a common temperature of 20°C), separated by experiment order (whether fish underwent thermal tolerance or hypoxia tolerance trials first). There were significant main effects of acclimation temperature and experiment order on measures of hypoxia tolerance but no significant interactions between acclimation temperature and experiment order (two-way ANOVA; see Results for details). All data is represented as mean ± S.E.M. (n = 4 fish per acclimation temperature and experiment order).

Acclimation temperature	Experiment order	ILOS (%)	Time in hypoxia (min)
14°C	CT_{max} then ILOS	16.17 ± 1.41	99.5 ± 36.0
	ILOS then CT_{max}	15.41 ± 0.71	113.0 ± 26.6
16°C	CT_{max} then ILOS	11.09 ± 2.25	418.0 ± 178.3
	ILOS then CT_{max}	9.98 ± 3.51	406.3 ± 180.5
18°C	CT_{max} then ILOS	13.09 ± 0.95	241.3 ± 63.9
	ILOS then CT_{max}	10.10 ± 1.50	500.0 ± 129.7
20°C	CT_{max} then ILOS	5.20 ± 3.10	703.8 ± 141.2
	ILOS then CT_{max}	0 ± 0	905 ± 0
22°C	CT_{max} then ILOS	7.85 ± 4.63	510.0 ± 230.1
	ILOS then CT_{max}	1.58 ± 1.58	890.8 ± 14.3
